# Structured neighboring-sarcomere switching is coupled to variable-reach internal length redistribution during hyperthermal sarcomeric oscillations in living cardiomyocytes

**DOI:** 10.2142/biophysico.bppb-v23.0024

**Published:** 2026-07-15

**Authors:** Seine A. Shintani

**Affiliations:** 1 Department of Biomedical Sciences, College of Life and Health Sciences, Chubu University, Kasugai, Aichi 487-8501, Japan; 2 Center for Mathematical Science and Artificial Intelligence, Chubu University, Kasugai, Aichi 487-8501, Japan; 3 Institute for Advanced Research, Nagoya University, Nagoya, Aichi 464-8601, Japan

**Keywords:** cardiomyocyte, sarcomere, hyperthermal sarcomeric oscillation, phase switch, length redistribution

## Abstract

Hyperthermal sarcomeric oscillations (HSOs) expose rapid sarcomere-level motion in living cardiomyocytes and provide a mesoscopic window between actomyosin activity and robust cellular contraction. I reanalyzed high-speed sarcomere-length recordings from five consecutive sarcomeres in each of seven neonatal rat cardiomyocytes. During HSOs, local phase relations became trackable through most of the oscillatory segment (valid fraction, 0.298 before warming and 0.956 during HSOs; paired Wilcoxon P=0.0156). Neighboring-sarcomere reconfiguration was dominated by one-link switches, in which one adjacent-pair relation changed while the other three were maintained (216/230 HSO phase transitions), and anti-phase-rich occupancy increased from 0.254 to 0.509 (P=0.0156). I then measured event-local relative internal length redistribution. For each reach-qualified one-link event, compensation reach, S, was defined as the expected sarcomere-index distance between relative shortening and relative lengthening. The same directed IAAI-to-IAII switch was accompanied by short-reach redistribution in one event (S=1.29) and cross-chain redistribution in another (S=2.88). Across 248 reach-qualified events, S increased with the pre-event number of I-type links, with a cell-fixed slope of 0.148 span units per added I-link supported by cell-blocked permutation and cell-cluster bootstrap analyses. Thus, HSO reveals a mesoscale organizing process in which a local switch in neighboring-sarcomere synchrony is linked to spatially distributed relative internal length redistribution whose reach is shaped by the pre-event phase context.

## Significance

The contractile rhythm of a cardiomyocyte emerges between stochastic molecular motors and whole-cell shortening. This study uses hyperthermal sarcomeric oscillations to resolve the intermediate scale formed by several connected sarcomeres. Neighboring-sarcomere phase relations reconfigure mainly through one-link switches, and the same switch is coupled to relative internal length redistribution over different spatial reaches. I-rich pre-event states are associated with broader redistribution reach. These findings show how rhythmic motion can stabilize local imbalance by linking synchrony changes to distributed sarcomere-length responses.

## Introduction

Cardiac contraction is generated by molecular motors, but the heartbeat is expressed as a robust cellular and organ-level rhythm. Cross-bridge theory and modern single-molecule studies have established how actomyosin motors generate force and motion, while lattice-scale models show that those molecular events occur inside a geometrically constrained sarcomere architecture [1–5]. Between these two levels lies a mesoscopic regime: several sarcomeres arranged in series, mechanically connected, and able to exchange length while preserving the rhythm of the cell. Resolving that regime is essential for understanding how stochastic molecular behavior is organized into stable cardiac motion.

Individual sarcomere measurements have made this mesoscopic level experimentally accessible. Sarcomere-length nanometry demonstrated nanometer-scale tracking of individual sarcomeres in neonatal cardiomyocytes and showed that cell-averaged traces mask sarcomere-to-sarcomere dynamics [6]. The same experimental direction connected local calcium signals and single-sarcomere length at matched spatial resolution [6,7]. Complementary studies in isolated cells and living myocardium have shown that sarcomere strain, synchrony, length variability, and asynchronous motion are organized features of cardiac mechanics [8–12]. These findings frame a direct question: when neighboring sarcomeres change their local timing relationship, how is the resulting length change distributed through the connected chain?

Hyperthermal sarcomeric oscillation provides a focused experimental state for that question. My colleagues and I discovered and named HSO as a rapid, Ca^2+^-independent sarcomeric auto-oscillation induced when living neonatal rat cardiomyocytes are heated in the approximately 38–42°C range; the fast component is typically approximately 5–10 Hz [13]. Subsequent studies showed that warmed cardiomyocytes preserve the HSO period while changing oscillation amplitude and that HSO waveforms contain nonlinear or chaotic signatures at the sarcomere level [14–16]. These observations point to a dynamic form of stability: the warmed sarcomere system maintains rhythm through sustained oscillatory motion. HSO therefore allows repeated observation of local sarcomere rephasing within a living contractile cell.

The present study asks how one local change in neighboring-sarcomere synchrony is expressed as a length response in the surrounding sarcomere chain. I describe each adjacent sarcomere pair as in-phase (I) or anti-phase (A) and follow the four-link word formed by five consecutive sarcomeres. A one-link switch is an elementary update in which one neighboring relation changes while the remaining three are preserved. I then measure how that switch is expressed in the continuous sarcomere-length signals by computing where relative shortening and relative lengthening are placed along the observed chain. This design directly connects local phase switching to internal length compensation.

The working hypothesis is that a one-link HSO phase switch acts as a local entry point for mesoscopic relative length redistribution. If this is correct, the same directed phase switch should appear with different redistribution reaches in different events, and the pre-event local phase context should influence how far relative shortening and relative lengthening are separated. The results support both predictions and identify compensation reach as a quantitative bridge between neighboring-sarcomere phase organization and sarcomere-length redistribution.

## Materials and methods

### Cell preparation and microscopic recording

The sarcomere-length recordings were obtained from cultured neonatal rat cardiomyocytes prepared under the Animal Experiment Implementation Manual of the University of Tokyo with approval from the Animal Experiment Committee of the Faculty of Science, The University of Tokyo, as described previously [13,14]. Wistar rats at postnatal day 1 were purchased from Sankyo Labo Service Corporation (Tokyo, Japan). Immature cardiomyocytes were isolated from one-day-old rats, cultured in DMEM/F-12 medium supplemented with 10% fetal bovine serum, 100 U/ml penicillin, and 100 U/ml streptomycin, and transfected with pAcGFP-actinin [6,13,14]. Cells were observed one day after transfection. Sex classification was omitted because cardiomyocytes were isolated from one-day-old suckling pups.

Recordings were acquired with a previously reported high-spatiotemporal-resolution heating microscope system for individual sarcomere motion [13,14]. Briefly, an inverted microscope (IX-70, Olympus) was equipped with a high-sensitivity camera (iXon3 860/iXon Ultra, Andor Technology) and a 100× oil-immersion objective lens (UPlanSapo 100XO, Olympus). The camera pixel size corresponded to approximately 150 nm at the sample plane, and the nominal frame rate was 500 frames/s for high-time-resolution analysis. The sample stage was maintained at 37.0±0.2°C, and a 1550-nm infrared laser was used to rapidly heat the region around the monitored cardiomyocyte. In the high-speed recordings reanalyzed here, the local temperature near the cell was raised from the 37°C stage condition to approximately 41°C, corresponding to an approximately 4°C local temperature jump. A 488-nm laser was used for AcGFP-α-actinin fluorescence excitation. Sarcomere length was measured from α-actinin-AcGFP Z-line intensity profiles by parabolic fitting, as described previously [6,13,14].

### Internal sarcomere-length coordinates

Five consecutive sarcomeres were analyzed in each of seven cells. Let L_c,i_(t) denote the length of sarcomere i=1,...,5 in cell c at time t. The instantaneous local mean was

(1) $${\bar{L}}_{c}\left(t\right)=\frac{1}{5}\sum_{i=1}^{5}L_{c,i}\left(t\right),$$

and the internal coordinate was

(2) $$X_{c,i}\left(t\right)=L_{c,i}\left(t\right)-{\bar{L}}_{c}\left(t\right).$$

This mean-subtracted coordinate measures length redistribution within the observed five-sarcomere chain. The HSO-band internal coordinate Y_c,i_(t) was obtained from X_c,i_(t) using a third-order Butterworth band-pass filter applied in a zero-phase forward-backward manner. The cell-specific passbands were determined from the dominant HSO frequency in the 9.5–17.5 s HSO segment, with a high-frequency candidate range of 3.5–25 Hz and a dominant-frequency search range of 4.5–9.5 Hz. The exact passbands used for each cell are provided in Supplementary Table S1.

### Neighboring-pair phase state and one-link switching

The analytic signal of each HSO-band internal trace was computed using the Hilbert transform. The instantaneous phase of sarcomere i was

(3) $$\Phi_{c,i}\left(t\right)=\arg\left[ℋ\{Y_{c,i}\left(t\right)\}\right],$$

where H denotes the analytic signal. For each neighboring pair k=(i,i+1), the phase difference was Δ φ_c,k_(t)=φ_c,i+1_(t)-φ_c,i_(t). A link was classified as in-phase (I) when cosΔφ_c,k_(t)≥0 and anti-phase (A) when cosΔφ_c,k_(t)<0. The four adjacent-pair labels form a four-letter local phase word, such as IAAI.

Phase-valid intervals were defined from the high-frequency component (HFC) envelope. For each cell, the HFC envelope was calculated over the full recording, and the high and low hysteresis thresholds were defined from fixed quantiles of that same full-recording envelope. The before-warming and HSO segments were therefore evaluated with the same cell-specific thresholds, providing a common reference for the two conditions. Short gaps were filled and short isolated intervals were removed with fixed duration criteria to suppress unstable state calls. Consecutive valid dwell states were then compared by Hamming distance. A one-link switch was defined as a transition in which exactly one of the four adjacent-pair labels changed and the other three were maintained. This definition makes the local phase update directly readable as a change in one neighboring-sarcomere relation.

The five-sarcomere window was chosen because the high-speed recordings provided six clear Z-line positions, yielding five consecutive sarcomere lengths, in each analyzed cell. This window contains four local links, which is the smallest line segment in the present dataset that supports a four-letter local state word and a spatial compensation metric with adjacent, intermediate, and cross-window placements. I therefore restrict all conclusions to the observed five-sarcomere chain.

### Compensation reach of one-link events

For each reach-qualified one-link HSO event, I summarized the HSO-band internal coordinate immediately before and after the update and computed the event-local internal change vector

(4) $$\Delta Y_{e}=Y_{e}^{+}-Y_{e}^{-},$$

where Y^–^_e_ and Y^+^_e_ are five-component pre-event and post-event summary vectors. The event time was defined as the boundary between two consecutive valid dwell states. Y^–^_e_ was computed as the component-wise median of Y_c,i_(t) over the final 0.08 s of the pre-event dwell state, or over the full pre-event dwell if it was shorter. Y^+^_e_ was computed as the component-wise median over the first 0.08 s of the post-event dwell state, or over the full post-event dwell if it was shorter. Events were retained only when both summary windows contained at least five samples. The median statistic was used to summarize the dwell-state level while reducing sensitivity to single-frame fluctuations at the transition boundary. Negative components of Δ Y_e_ were treated as relative shortening and positive components as relative lengthening. The normalized shortening and lengthening weights were

(5) $$p_{e,i}^{-}=\frac{\max\left(-\Delta Y_{e,i},0\right)}{\sum_{m=1}^{5}\max\left(-\Delta Y_{e,m},0\right)},\;\;p_{e,j}^{+}=\frac{\max\left(\Delta Y_{e,j},0\right)}{\sum_{m=1}^{5}\max\left(\Delta Y_{e,m},0\right)}.$$

The compensation reach was defined as

(6) $$S_{e}=\sum_{i=1}^{5}\sum_{j=1}^{5}p_{e,i}^{-}p_{e,j}^{+}\left|i-j\right|.$$

In practical terms, S measures how many sarcomere spacings separate the relative-shortening side from the relative-lengthening side of the same local phase-switch event. I interpret S as a measured reach of relative internal length redistribution associated with the switch. Events with nonzero shortening and lengthening weights were included in the reach analysis.

The topology and compensation analyses used purpose-specific event populations derived from the same seven cells. For the local phase-topology analysis, I used 230 valid dwell-to-dwell HSO phase transitions, including 216 one-link transitions, to quantify how neighboring-pair phase states were updated during HSO. For the internal redistribution analysis, I used 248 reach-qualified one-link HSO events from event-local update windows in which the post-minus-pre internal change vector contained both relative shortening and relative lengthening, allowing compensation reach S to be calculated. These two event sets therefore have different counts by design. The topology population asks how neighboring-pair phase states switch, whereas the reach-qualified population asks how each measurable one-link switch is expressed as relative internal length redistribution.

### Robustness checks and statistics

The local phase-topology analysis used before-warming and HSO segments from the same seven cells. Paired before-warming versus HSO comparisons used the Wilcoxon signed-rank test. To test the stability of the topology results, I performed two robustness checks. First, the one-link fraction was recomputed across alternative gate and bandwidth settings (Supplementary Table S2). Second, anti-phase-rich occupancy was recomputed after estimating phases from raw HSO-band sarcomere-length traces without instantaneous local-mean subtraction; the median per-cell anti-phase-rich fraction increased from 0.111 before warming to 0.460 during HSOs under this alternative preprocessing (Supplementary Table S3). These checks support the topology interpretation within the measured five-sarcomere window.

The compensation-reach analysis used 248 reach-qualified one-link HSO events from the same seven cells. The association between pre-event I-link count

(7) $$M_{I,e}=\sum_{k=1}^{4}b_{e,k}^{-},$$

where b^–^_e,k_=1 for I and 0 for A in the pre-event state, and compensation reach was summarized by a cell-fixed linear slope. Cell-blocked permutation shuffled M_I_ labels within each cell, preserving cell-level event structure while breaking the event-level pairing between M_I_ and S. Cell-cluster bootstrap resampled cells with replacement to summarize the stability of the slope.

## Results

### HSO reveals sustained internal sarcomere oscillation within a beating cardiomyocyte

**Figure 1:**
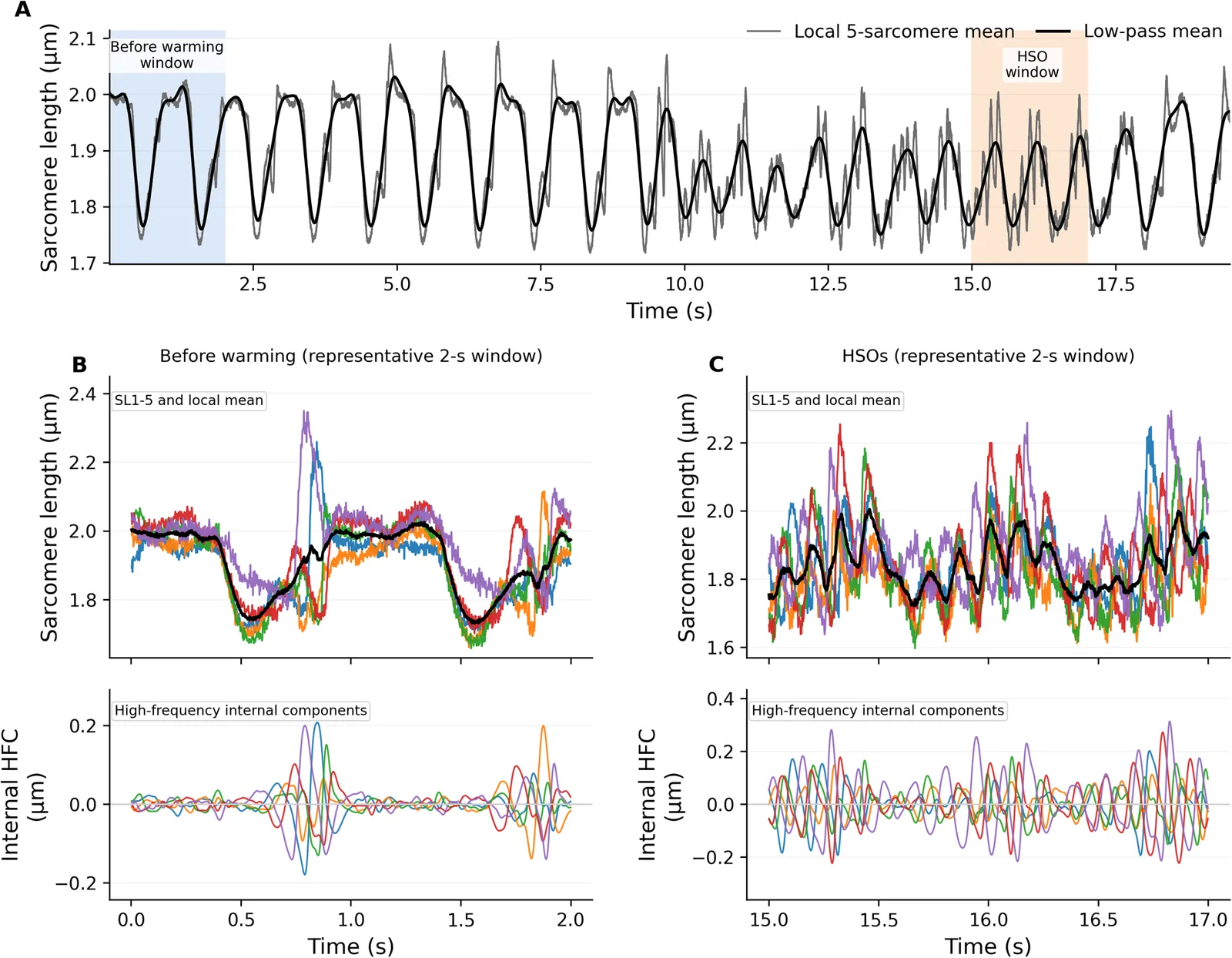
Primary sarcomere-length time-series context before warming and during HSOs. A, Full-trace local five-sarcomere mean trace for an exemplar cell, with a low-pass mean overlaid. Shaded windows indicate the before-warming and HSO segments shown below. B, Representative before-warming window. The upper panel shows sarcomere-length traces of five consecutive sarcomeres and their local mean; the lower panel shows high-frequency internal components after subtraction of the instantaneous local mean. C, Representative HSO window shown in the same format. During HSO, the beat-scale envelope remains visible while fast internal sarcomere oscillations become sustained.

The raw sarcomere-length traces show how warming changes the internal dynamics of the observed five-sarcomere segment. Before warming, the local mean trace is dominated by the beat-scale envelope, and fast internal oscillations appear intermittently. During HSO, the beat-scale rhythm remains visible while the five sarcomeres exhibit sustained rapid internal oscillations superimposed on that rhythm (Figure 1A–C). This pattern motivates the central analysis: the cell preserves a recognizable contractile rhythm while neighboring sarcomeres reorganize their local timing and length redistribution within the same segment.

### Local phase relations become trackable during HSO

**Figure 2:**
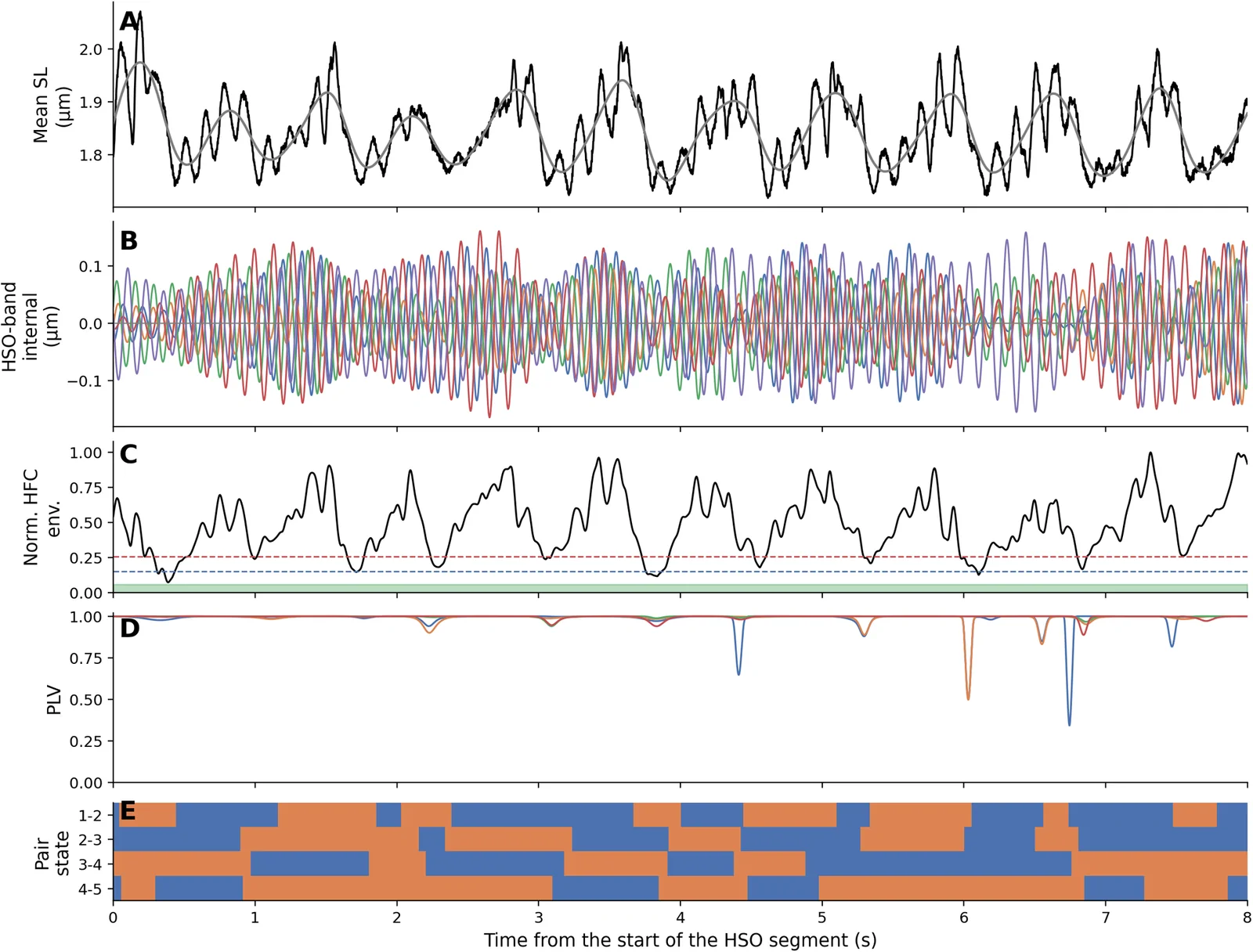
Definition of the local phase state during a representative HSO segment. A, Local five-sarcomere mean trace and its low-pass component. B, HSO-band internal signals for the five sarcomeres. C, Normalized high-frequency envelope and hysteresis thresholds that define the phase-valid gate; the upper and lower dashed lines mark the high and low thresholds of the hysteresis gate. D, Phase-locking value for the four neighboring pairs. E, Binary neighboring-pair states for pairs 1–2, 2–3, 3–4, and 4–5. Blue indicates in-phase (I), and orange indicates anti-phase (A). The segment is shown with the refined visualization gate for display continuity; quantitative statistics use the fixed primary gate.

I next quantified adjacent-pair phase relations in the HSO-band internal signals. Figure 2 shows the analysis path for a representative HSO segment: the local mean and low-pass envelope, the HSO-band internal traces, the normalized high-frequency envelope used for gating, the phase-locking values of the four neighboring pairs, and the binary I/A state traces for links 1–2, 2–3, 3–4, and 4–5 (Figure 2A–E). The colored dashed lines in Figure 2C indicate the high and low hysteresis thresholds used for the phase-valid gate. This representation provides the local state vocabulary used to quantify neighboring-sarcomere phase updates during HSO.

### Neighboring-sarcomere reconfiguration proceeds mainly through one-link switches

**Figure 3:**
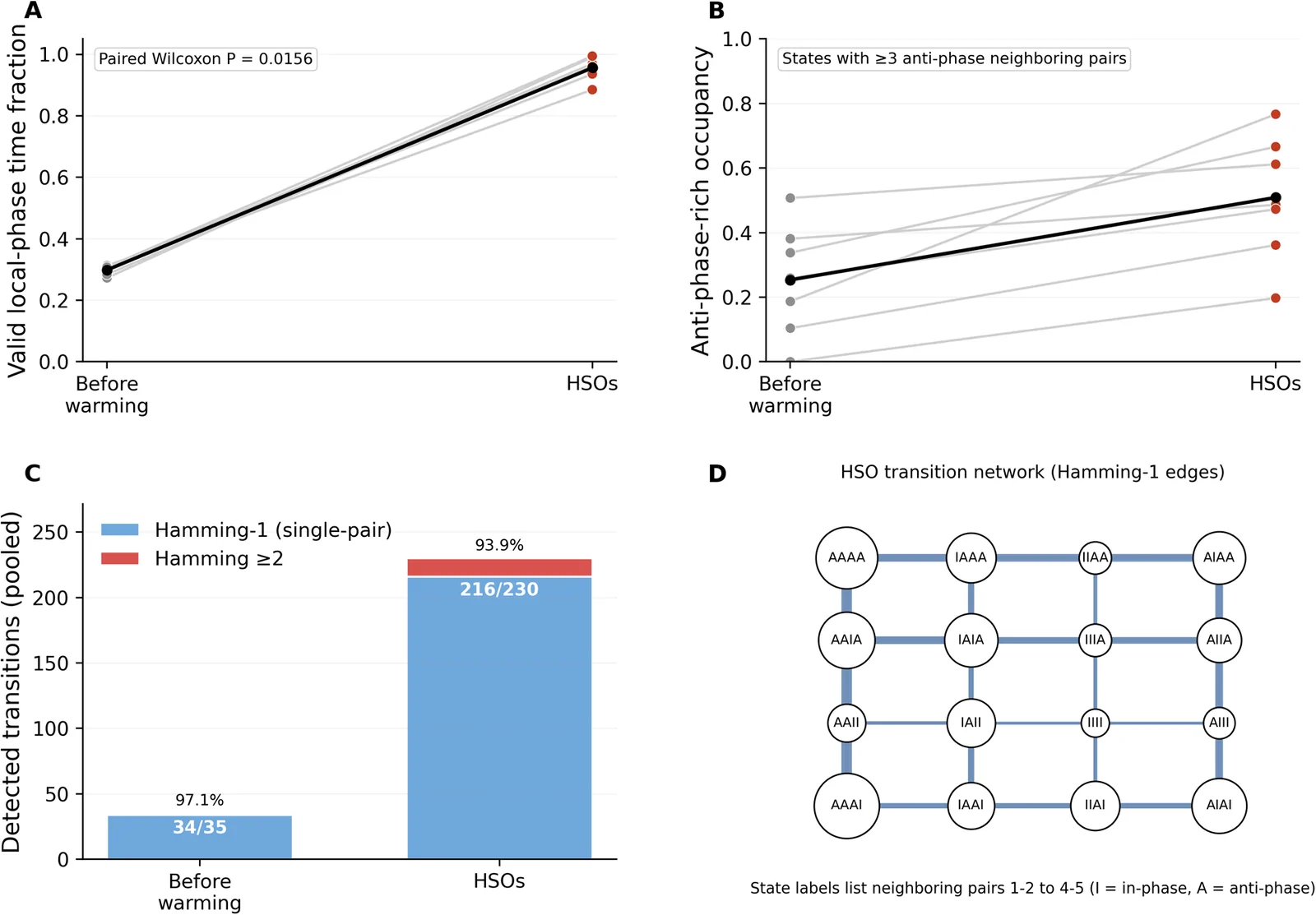
HSOs reveal a constrained local phase topology. A, Paired values for the fraction of time in which local phase tracking was valid before warming and during HSO. B, Paired values for time spent in anti-phase-rich states, defined as states with three or more anti-phase neighboring pairs. C, Pooled transition topology before warming and during HSO. The HSO phase-topology analysis used 230 valid dwell-to-dwell HSO phase transitions from seven cells, including 216 one-link transitions. Numbers inside the bars indicate the count of one-link transitions relative to all detected transitions. A one-link transition means that only one of the four neighboring-pair relations changed between successive patterns. D, HSO transition network restricted to one-link edges. State labels encode the four neighboring pairs from 1–2 to 4–5, with I for in-phase and A for anti-phase. Edge width scales with pooled HSO transition count.

The same analysis showed that local phase tracking became robust during HSO: the valid fraction increased from 0.298 before warming to 0.956 during HSO (paired Wilcoxon P=0.0156; Figure 3A). At each valid time point, the five-sarcomere segment was represented by a four-letter word describing the I/A relation of the four neighboring pairs. HSO reconfiguration moved through this 16-state space in a highly constrained manner. During HSO, 216 of 230 detected transitions were one-link transitions, corresponding to 93.9% of HSO transitions (Figure 3C). Every cell retained a high HSO one-link fraction in the cell-wise analysis, and the one-link fraction remained high across alternative gate and band-width settings (Supplementary Table S2). These results identify one-link switching as the dominant elementary update of neighboring-sarcomere phase organization during HSO.

The state occupancy also shifted. The fraction of valid time spent in anti-phase-rich states, defined as states with three or more anti-phase neighboring pairs, increased from 0.254 before warming to 0.509 during HSO (paired Wilcoxon P=0.0156; Figure 3B). The same direction of change was retained when phases were estimated from raw HSO-band traces without local-mean subtraction (Supplementary Table S3). The HSO transition network restricted to one-link edges shows how these elementary switches connect the 16 local phase words (Figure 3D). Thus, HSO organizes neighboring sarcomeres through stepwise switching among local synchrony and anti-phase synchrony states.

### The same one-link switch is coupled to different internal redistribution reaches

The one-link result defines the elementary update of local synchrony or anti-phase synchrony. The next question is how the connected sarcomere chain expresses that update as relative internal length motion. I therefore measured the event-local internal change vector for one-link HSO events and decomposed it into relative shortening and relative lengthening components.

**Figure 4:**
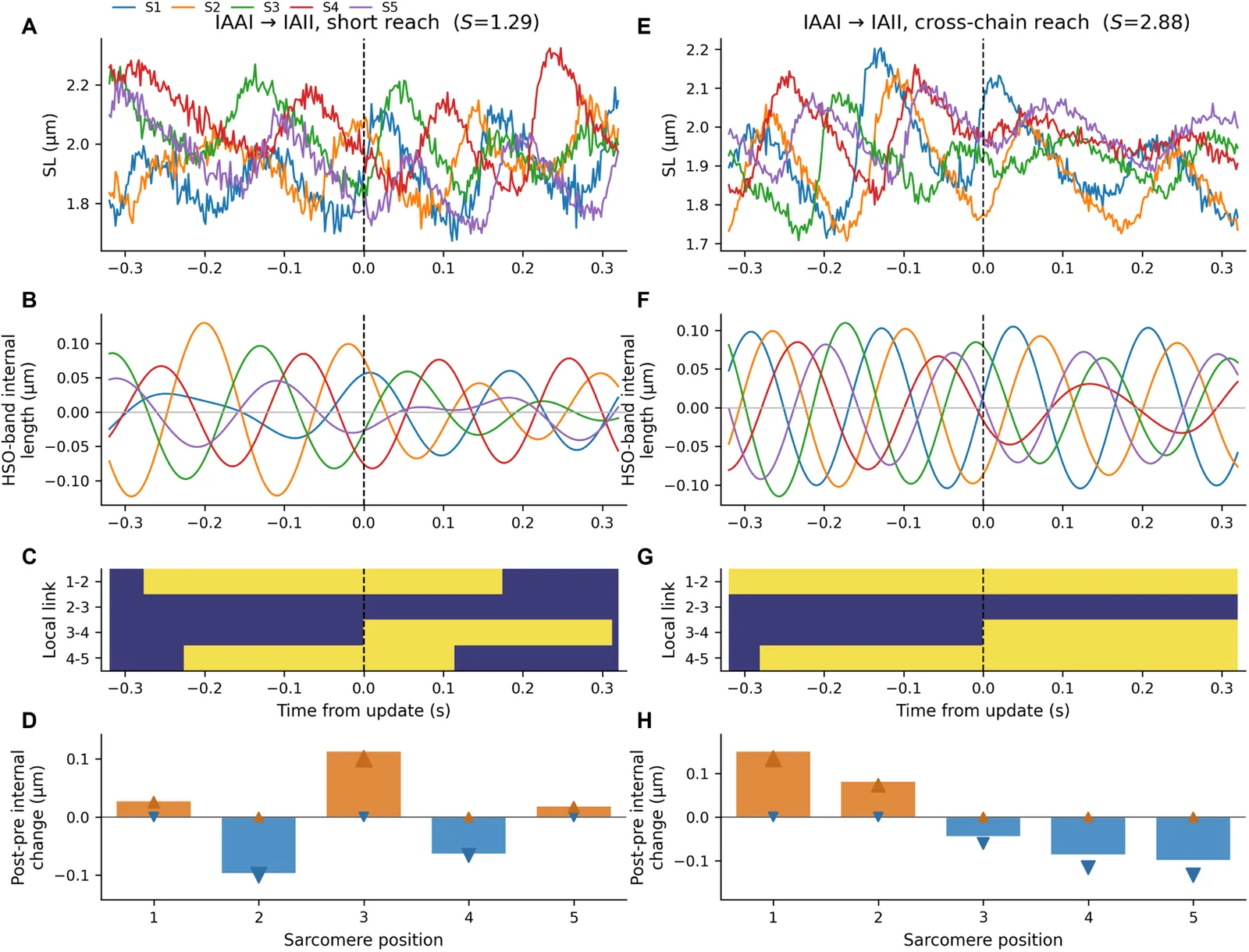
The same IAAI-to-IAII phase switch is coupled to different internal redistribution reaches. A–D, Short-reach example. A, Raw sarcomere lengths of five consecutive sarcomeres. B, HSO-band internal length traces after subtraction of the five-sarcomere mean. C, Binary I/A state of the four local links; the dashed line marks the update time. D, Post-minus-pre internal change vector. Orange bars indicate relative lengthening, and blue bars indicate relative shortening; the compensation reach is S=1.29. E–H, Cross-chain example with the same directed IAAI-to-IAII switch. E, Raw sarcomere lengths. F, HSO-band internal length traces. G, Local-link I/A state. H, Post-minus-pre internal change vector. Relative lengthening and shortening are placed across a wider part of the observed chain, giving S=2.88. Cross-chain refers to the observed five-sarcomere chain.

Two representative events with the same directed IAAI-to-IAII switch illustrate the result. In the short-reach example, the raw sarcomere-length traces first show the measured five-sarcomere motion, the HSO-band internal traces isolate rapid internal redistribution, and the local-link map marks the IAAI-to-IAII update (Figure 4A–C). The post-minus-pre internal change vector places the major shortening and lengthening components close to one another, giving S=1.29 (Figure 4D). In the cross-chain example, the same sequence of raw trace, HSO-band trace, and local-link map shows the same directed IAAI-to-IAII update (Figure 4E–G). Its change vector places relative lengthening near the beginning of the chain and relative shortening toward the distal part of the observed chain, giving S=2.88 (Figure 4H). The same elementary phase switch is therefore coupled to short-reach or cross-chain relative internal length redistribution within the five-sarcomere window.

### I- rich pre-event local states are associated with broader compensation reach

**Figure 5:**
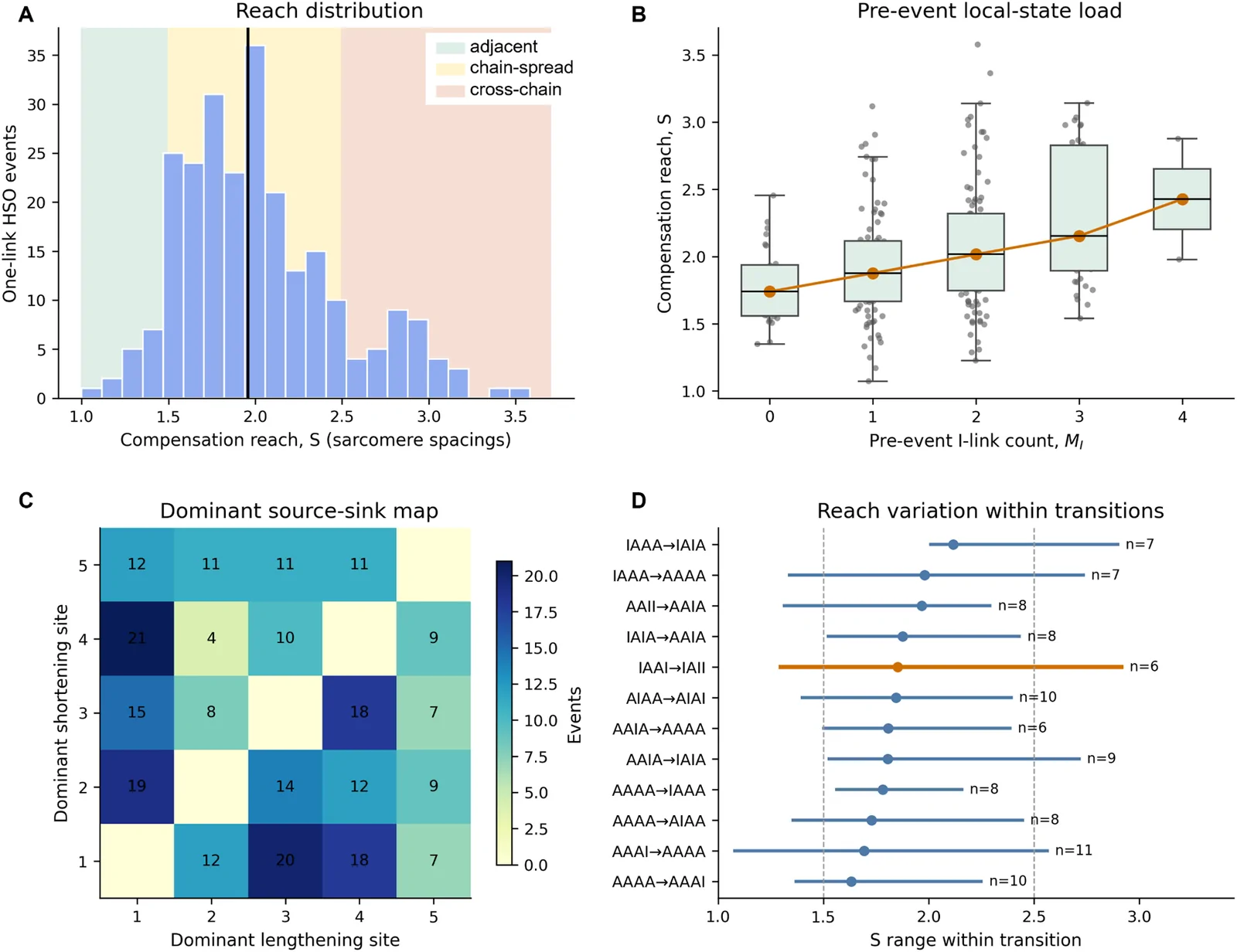
Compensation reach measures a population-level axis that expands in I-rich pre-event states. A, Distribution of compensation reach, S, across 248 reach-qualified one-link HSO events from seven cells. Events were included when the post-minus-pre internal change vector contained both relative shortening and relative lengthening, allowing S to be calculated. The background bands show adjacent-reach, chain-spread, and cross-chain regimes. The vertical line marks the median. B, Compensation reach grouped by the pre-event I-link count, MI. Points represent events, box plots summarize group distributions, and the orange line links group medians. C, Dominant source-sink map showing the dominant relative shortening site and dominant relative lengthening site. D, Reach variation within major directed transitions. Points indicate median S and horizontal lines indicate the range within each transition.

The representative examples indicate that one-link switching has a measurable redistribution reach. I next quantified this reach across the event population. Across 248 reach-qualified one-link HSO events, S formed a continuous distribution with median 1.958 sarcomere spacings (interquartile range, 1.690–2.268). Using descriptive regimes on this single reach axis, 18 events were adjacent-reach, 192 were chain-spread, and 38 were cross-chain (Figure 5A).

The reach distribution was organized by pre-event phase context. The median S increased as the pre-event I-link count M_I_ increased: 1.739 for M_I_=0, 1.876 for M_I_=1, 2.016 for M_I_=2, 2.153 for M_I_=3, and 2.427 for M_I_=4. The cell-fixed slope was 0.148 span units per added I-link (Figure 5B). The source-sink map shows that dominant shortening and lengthening sites are distributed throughout the observed chain rather than concentrated in a single anatomical pair (Figure 5C). Major directed transitions also occupied a range of S values, including the highlighted IAAI-to-IAII transition (Figure 5D). These results show that I-rich local states are associated with broader relative internal length redistribution during one-link HSO switching.

### Cell-aware and coordinate controls support the reach interpretation

**Figure 6:**
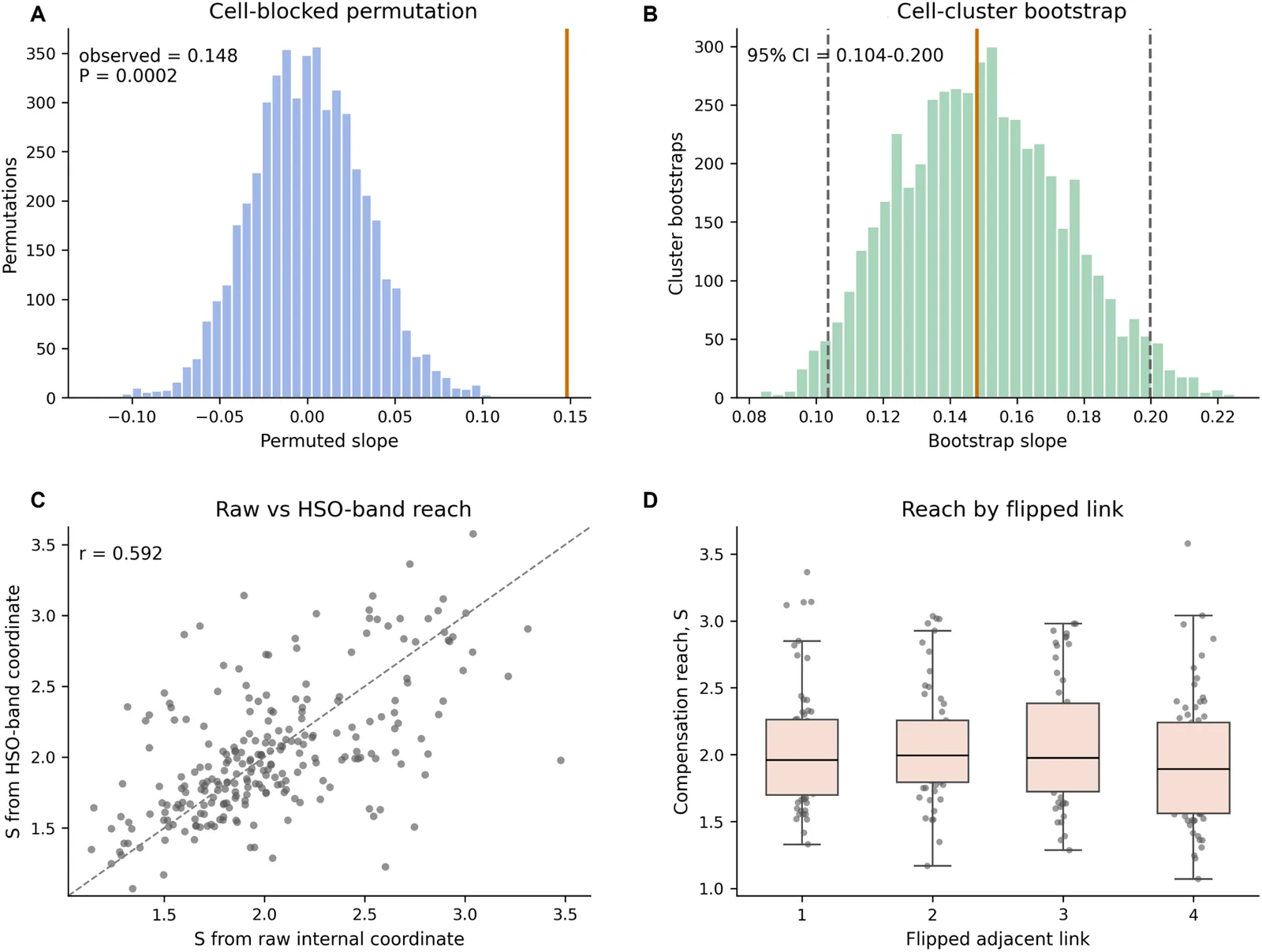
Cell-aware and coordinate controls for compensation reach. Unless otherwise indicated, controls used the 248 reach-qualified one-link HSO events used for the compensation-reach analysis. A, Cell-blocked permutation analysis. Pre-event I-link counts were shuffled within each cell, and the slope linking MI to S was recomputed. The orange line marks the observed slope. B, Cell-cluster bootstrap analysis. Cells were resampled with replacement, and the dashed lines show the 95% bootstrap interval for the slope. C, Comparison of compensation reach computed from raw internal coordinates and HSO-band internal coordinates. The positive correlation indicates that HSO-band reach tracks length redistribution present in the measured internal coordinate. D, Compensation reach grouped by the flipped adjacent link. The overlapping distributions and flipped-link-adjusted slope show that the pre-event I-link load is associated with reach across switched-pair positions.

The reach association was evaluated with controls designed around likely alternative explanations. First, cell-blocked permutation testing shuffled M_I_ labels within each cell. This keeps cell-level event structure intact while breaking the event-level pairing between pre-event state and reach. The observed slope of 0.148 lay at the positive edge of the null distribution, giving P=0.0002 (Figure 6A). Second, cell-cluster bootstrap resampling produced a 95% interval of 0.104–0.200 for the slope, indicating that the positive association was stable when cells, rather than individual events alone, were used as the resampling unit (Figure 6B).

The reach metric also tracked measured length redistribution beyond the filtered coordinate used for the main phase analysis. Compensation reach computed from HSO-band internal coordinates was positively associated with reach computed from raw internal coordinates (Pearson r=0.592; Figure 6C). Finally, grouping events by the flipped adjacent link showed overlapping reach distributions, and the M_I_ slope remained positive after flipped-link adjustment (adjusted slope, 0.147 span units per I-link; cell-blocked permutation P=0.0005; Figure 6D). These controls support the interpretation that compensation reach reflects event-local internal length redistribution shaped by pre-event phase context.

## Discussion

This study identifies a mesoscopic organizing process during HSO. Neighboring sarcomeres rephase mainly by one-link switches, and those switches are accompanied by relative internal length redistribution with variable spatial reach. In practical terms, one adjacent sarcomere pair changes its synchrony or anti-phase synchrony relation, and the connected five-sarcomere chain organizes where relative shortening and relative lengthening are placed. HSO therefore reveals how a local rephasing event is expressed as a distributed length response within a living cardiomyocyte.

The one-link switching result is important because it defines an elementary sarcomere-unit update. During HSO, the local phase word moves through the 16-state space primarily by changing one neighboring relation at a time. This provides a direct mesoscopic description of sarcomere coordination: the operative reorganization unit is a neighboring-sarcomere link embedded in a short connected chain (Figure 3C, D).

The compensation analysis adds the metric counterpart to this topology. A switch that is elementary in the phase-state map can be expressed as short-reach, chain-spread, or cross-chain redistribution in length space. The representative IAAI-to-IAII events show this point directly, and the population analysis shows that it is part of a graded reach axis across events (Figures 4 and 5A). The positive relationship between M_I_ and S gives the axis biological meaning: I-rich pre-event states are contexts in which a local phase switch is accompanied by broader relative internal length redistribution (Figure 5B).

HSO is best viewed as an oscillatory dynamic state of a warmed sarcomere system. The original HSO experiments showed that rapid heating of living neonatal cardiomyocytes induces fast, largely Ca^2+^-independent sarcomeric auto-oscillations [13,14]. Prior work supports a simple mechanistic picture with two coupled layers. First, warming moves the contractile apparatus toward an intermediate activation state: microscopic heat pulses activate cardiac thin filaments by shifting tropomyosin regulation and facilitating myosin binding [17]. Second, strain-dependent actomyosin cycling, elastic coupling, and sarcomere-to-sarcomere communication convert molecular cycling into self-sustained oscillation and traveling waves [18,19]. In this picture, temperature opens the oscillation-permissive state, and rhythmic shortening-lengthening redistribution is the stable output of the mechanically connected sarcomere chain.

Calcium remains an important partner variable for HSO mechanism, and the present length-based analysis can be placed in that calcium context. In the original HSO discovery study, sarcomere motion and intracellular calcium were monitored together, and the calcium signal was observed as a cell-level change on the beat-scale, whereas the rapid HSO component appeared in sarcomere length [13]. Local calcium and single-sarcomere length can also be imaged simultaneously in neonatal cardiomyocytes [7], and later HSO work showed that slow calcium-dependent contractions can coexist with fast, largely Ca^2+^-independent HSO components [14]. The high-speed 500 frames/s dataset analyzed here was designed to resolve sarcomere-length dynamics with high temporal precision. This focus directly supports the present analysis of one-link phase switching and relative internal length redistribution. A future experiment combining high-speed sarcomere tracking with local calcium imaging would add the next mechanistic layer by testing whether small local calcium fluctuations modulate I/A-state lifetimes or switching probability.

The striated-muscle comparison strengthens this physical picture. HSO was originally observed and named in primary cultured neonatal cardiomyocytes [13]. Related thermally sensitive sarcomere oscillations have also been analyzed in rabbit skeletal myofibrils, where microscopic heating induced sarcomeric auto-oscillations in pressure-perturbed myofibrils and revealed the importance of passive elastic properties for communication between adjacent sarcomeres [20]. In skinned cardiac and skeletal muscle preparations, spontaneous oscillatory contraction and inter-sarcomere coordination after quick stretch or phosphate jumps further demonstrate that sarcomeres can coordinate through mechanical coupling across muscle systems [21–27]. The present conclusions are drawn from living neonatal cardiomyocytes, and the broader comparison suggests a common principle: warming, thin-filament regulation, cross-bridge kinetics, passive elasticity, and cellular architecture can together place striated sarcomeres into coordinated oscillatory states.

The structural basis of rephasing is best described as a distributed sarcomere circuit. Z-disc alignment, serial sarcomere geometry, titin-based passive elasticity, myofibrillar connectivity, thin-filament regulatory state, and cross-bridge kinetics act in the same mechanically connected lattice. This organization matters because the measured response has two different dimensions: the one-link switch is a change in one adjacent-pair phase relation, whereas the compensation vector is a five-sarcomere source-sink pattern. The same IAAI-to-IAII switch can be expressed as short-reach redistribution or cross-chain redistribution, and the reach varies with pre-event phase context (Figures 4 and 5). A single isolated anatomical element can contribute to this behavior, but the present length recordings resolve the integrated mesoscopic output of the connected sarcomere circuit. Selective perturbation or simultaneous structural readout will be required to assign individual contributions to particular elements.

Rapid local heating was used to create a reversible and well-timed thermal perturbation so that the onset of HSO could be related to the applied heat stimulus. In the original discovery study, a larger microscopic temperature jump of approximately 15°C was used to establish HSO induction [13], whereas the high-speed 500 frames/s recordings reanalyzed here used an approximately 4°C jump from the 37°C stage condition to about 41°C [14]. The rapid jump is therefore an experimental strategy for defining causal timing and obtaining analyzable HSO episodes, rather than a claim that an instantaneous jump is the only path into oscillation. A slow rise to the same final temperature may introduce additional adaptation processes, including changes in baseline calcium handling, and could alter the entry path into oscillation. Slow warming, steady incubation at elevated temperature, and rapid cooling are valuable future protocols for testing onset path, hysteresis, and the persistence of one-link switching after the thermal stimulus is removed, but they address a different dynamical question from the event-local structure resolved here.

Anti-phase-rich organization offers a practical sarcomere-level strategy for distributing internal strain. Alternating neighboring relations place shortening in one part of the chain against lengthening or reduced shortening elsewhere, allowing the observed chain to absorb local length imbalance while preserving a global rhythm. This interpretation aligns with sarcomere strain harmonization, sarcomere-length variability, and asynchronous sarcomere movement observed in cardiac muscle [8–12].

The measurement defines the scale of the conclusion. Cross-chain compensation denotes redistribution across the observed five-sarcomere chain. The I/A state provides a reproducible descriptor of adjacent-pair phase relation, and the compensation vector is computed from continuous sarcomere-length signals. The dataset is event-rich and cell-limited; therefore, the reach inference uses cell-blocked permutation and cell-cluster bootstrap controls (Figure 6A, B). Larger fields of view, longer sarcomere chains, and simultaneous measurements of local calcium and mechanical readouts will extend this framework toward whole-cell and tissue-level coordination.

The core conclusion is that HSO exposes a sarcomere-level grammar of local rephasing and length compensation. One-link switches define the elementary phase updates. Compensation reach defines how the connected chain distributes length in response to those updates. Together, these two coordinates identify a mesoscopic layer of cardiac organization between stochastic molecular motors and the averaged rhythm of the cardiomyocyte.

## Conclusion

Hyperthermal sarcomeric oscillations reveal a mesoscopic grammar of cardiomyocyte dynamics. Neighboring sarcomeres reconfigure mainly through one-link switches, and those switches are coupled to relative internal length redistribution over variable spatial reaches. I-rich pre-event local states are associated with broader compensation reach, linking the phase context of a local sarcomere chain to the spatial placement of length redistribution. This framework shows how rhythmic sarcomere motion links local synchrony changes to organized internal length balance.

## Conflict of interest

The author declares no competing interests.

## Author contributions

Seine A. Shintani designed the research, performed the experiments, analyzed data, prepared the figures, and wrote the manuscript.

## Data availability

The reanalyzed data supporting the findings of this study are available from the corresponding author upon reasonable request.

Preliminary versions of the local phase-topology analysis and the related internal-compensation analysis were deposited on bioRxiv (https://doi.org/10.64898/2026.03.13.711515 on April 1, 2026, and https://doi.org/10.64898/2026.03.26.714639 on April 4, 2026). The present revised manuscript consolidates these analyses into a single manuscript centered on one-link neighboring-sarcomere switching and internal length compensation.
